# Impact of an 8-Week Exercise and Sport Intervention on Post-Traumatic Stress Disorder Symptoms, Mental Health, and Physical Fitness among Male Refugees Living in a Greek Refugee Camp

**DOI:** 10.3390/ijerph16203904

**Published:** 2019-10-15

**Authors:** Florian Knappe, Flora Colledge, Markus Gerber

**Affiliations:** Department of Sport, Exercise and Health, University of Basel, 4052 Basel, Switzerland; florian.knappe@stud.unibas.ch (F.K.); Flora.colledge@unibas.ch (F.C.)

**Keywords:** fitness, Greece, mental health, physical activity, psychiatric symptoms, refugees

## Abstract

**Objective:** To explore the potential impact of exercise and sport training on symptoms of post-traumatic stress disorder (PTSD), depression, anxiety, quality of life, pain, and fitness in male refugees living in a Greek refugee camp. **Methods:** This investigation was designed as a one group pre-test/post-test study. A total of 45 refugees (*M_age_* = 25.6) participated in the data assessment. All participants were invited to engage in an 8-week exercise and sport intervention. Data were analysed with hierarchical regression analyses. **Results:** Baseline scores significantly predicted post-intervention scores across all study variables. Regression analyses showed that a higher participation rate predicted fewer anxiety symptoms, better health-related quality of life, higher self-perceived fitness, higher handgrip strength, and better cardiovascular fitness at post-intervention. A non-significant trend was also found for PTSD and depressive symptoms, showing that a higher participation rate was associated with fewer complaints at post-intervention. **Conclusions:** Among male refugees living in precarious conditions in a Greek refugee camp, frequency of participation in an 8-week exercise and sport training program seemed to have the potential to positively impact refugees’ health. Due to the pre-experimental study design, our results must be interpreted with caution.

## 1. Introduction

Since 2015, for the first time worldwide, more people are fleeing their home regions due to violence, hunger, and misery than after the end of the Second World War. More than 65 million people have been displaced from their homes [1]. As a result of political and social conflicts, as well as climate change, these figures are likely to increase.

Many refugees are subject to severe mental and physical strain (war, violence, political and religious persecution, poverty, imprisonment, torture) and often experience a traumatic flight (hard living conditions, economic hardship, discrimination, social exclusion, exploitation) [2]. According to Madsen et al. [3], almost half of all asylum seekers in Denmark were tortured and more than two thirds met the ICD-10 (International Classification of Diseases, 10th edition) criteria for post-traumatic stress disorder (PTSD). Upon arrival in the destination country, the “escape” is far from complete. Many of those affected spend several months or years in a refugee camp. Due to an uncertain residence status, the victims are plagued by future and existential fears. In addition, there is a high acculturative pressure on the refugees. The loss of homes, worries, or longing for family members and friends left behind are further post-migratory stressors. Unsurprisingly, in this context, refugees have a higher prevalence rate for psychopathological disorders such as PTSD, depression, or anxiety disorders [4]. These mental impairments are often accompanied by somatoform discomfort and pain [5]. Despite these findings, in the field of psychiatry, little attention has yet been paid to the treatment of refugees who suffer from PTSD symptoms [6].

Exercise and sport are thought to show promise in the treatment of PTSD symptoms, depression, and anxiety disorders. Exercise and sport activities are expected to promote individual resources, strengthen physical health, prevent conflict, promote integration, and improve individual wellbeing [7]. As highlighted by Korsik et al. [7], the United Nations High Commissioner for Refugees (UNHCR) recognizes the potential of exercise and sport and uses it in refugee camps partly as a peace-building measure. By contrast, contributing to the reduction of PTSD symptoms by means of exercise and sport is a relatively new goal, which is pursued (if at all) primarily by non-governmental organizations (NGOs). However, an empirical basis for assessing the effectiveness of exercise and sport-related measures has so far been largely absent.

In 2010, Lawrence et al. [8] published the results of their Cochrane review, in which they intended to summarize the state of research (up to 2008) on the effectiveness of sports and games in alleviating and/or diminishing the symptoms of PTSD in comparison to usual care or other interventions. Nevertheless, although the authors identified five potential papers, none of them met the inclusion criteria (participants with diagnosed PTSD, randomized controlled trials with one or more well-specified sports or games). Five years later, evidence of the potential benefits of exercise and sport interventions among traumatized individuals was provided in a meta-analysis by Rosenbaum et al. [9]. The findings of four randomized control trials (*n* = 200 participants) showed that such interventions are significantly more effective than control conditions for the reduction of PTSD and depressive symptoms. On the basis of these reviews, we can conclude that more controlled trials are needed to better and more reliably document the impact of exercise and sport interventions among individuals suffering from post-traumatic stress symptoms, and to optimize the use of such programs in specific target populations.

There are several reasons for further investigating the effect of exercise and sport interventions among refugees. First, international refugees have a significantly increased risk of suffering from psychiatric disorders such as PTSD, major depression, anxiety disorders, and insomnia compared to the general population, due to exposure to multiple stress factors [10,11].

Second, low levels of physical activity have been reported in several immigrant and refugee populations [12,13]. It is believed that the increased level of physical inactivity is due to apathy or resignation from previous traumatic experiences, prolonged asylum processes, feelings of hope- and helplessness, and the fear of deportation [14]. Liedl et al. [15] also point out that life conditions perceived as insecure and uncontrollable can lead to somatoform disorders such as painful muscle tensions, which present a barrier to regular physical activity for those affected. As a result, certain public health measures in host countries aim to understand the barriers to exercise and sport of refugees [16] and to increase their physical activity levels [17].

Third, patients with diagnosed PTSD have been shown to have an increased risk of cardiovascular and metabolic diseases and osteoporosis [18]. According to the Mutual Maintenance Theory by Sharp and Harvey [5], chronic pain and PTSD symptoms both contribute to the maintenance of these diseases. This double burden of mental stress and co-morbid physical illnesses may explain why psychiatric populations have a significantly lower life expectancy compared to “healthy” controls [19]. However, this life expectancy gap may also be due to increased substance abuse, especially among male refugees [20], which is particularly evident in refugee camps.

Abu-Saleh and Hughes [21], in a recent article on the mental health status of Syrian refugees, emphasized the need for greater efforts to improve the mental health of refugee populations. In doing so, they suggest that the measures should go beyond clinical therapy offerings, paying particular attention to non-clinical interventions that can promote health and individual resources. This is in line with a report by Médecins Sans Frontières [22], identifying a lack of early interventions as a key issue. Further, the findings of Brucks [23] indicate that asylum seekers manifest untreated mental disorders that are difficult to address the longer they go untreated; hence, organised approaches in the earliest stages of asylum-seeking are urgently required. As mentioned previously, very limited evidence is available regarding the effects of exercise and sport as part of the treatment of refugees suffering from high levels of PTSD symptoms. Not surprisingly, therefore, no national or international guidelines exist with regard to this topic [24]. Nevertheless, the few existing studies involving refugee populations suggest that exercise, sport, and physical activity interventions may have positive effects on various outcomes. For instance, Liedl et al. [15] found that a 12-week biofeedback-based cognitive behavioural therapy (CBT-BF) combined with physical activity (mixed activities, including endurance, strength, and flexibility training) produced better results compared to CBT-BF alone or a waiting-list control condition. In other words, participants of the CBT-BF with physical activity group showed an increased capacity to cope with pain after the intervention. Furthermore, high acceptability, compliance, and satisfaction was found for basic body awareness therapy (BBAT) among refugees living in Denmark who were suffering from trauma-related mental health problems [25]. Importantly, the refugees found BBAT helpful to relieve pain and tension, bring peace of mind and body, and make it easier to sleep [3]. Xin et al. [2] showed that a 12-week regular physical activity program had positive effects on the mental health of Bosnian refugees living in the USA. However, the control condition (general information about strategies useful for improving mental health) also made a positive contribution to the overall mental health of the refugees, so that the differences between the intervention and control group were not statistically significant at the post-test measurement.

In summary, while several studies have been carried out in countries of asylum, little research has been implemented with refugees (suffering from PTSD symptoms) in a camp setting to find out whether increased engagement in exercise and sport activities has positive effects on their mental health. Because these are very different contexts, a new focus on refugees living in a camp setting seems justified. Moreover, the existing studies in the countries of asylum have focused on body awareness therapy (including mainly light-intensity physical activities) as an add-on to standard care, whereas research on exercise and sport programs is missing.

The present study is the first investigation of whether an 8-week exercise and sport program among male refugees living in a Greek refugee camp has a positive impact on participants’ PTSD symptoms (primary outcome) and further health parameters such as depressive symptoms, anxiety symptoms, sleep complaints, pain, quality of life, as well as self-perceived and objectively assessed fitness. On the basis of previous research with healthy samples and populations suffering from PTSD symptoms, our expectation was that both the primary outcome (symptoms of post-traumatic stress [9]) and the secondary outcomes (mental health and fitness [26,27]) would improve among participants who regularly participate in exercise and sport activities. This is a novel line of study, as it (a) adds to the current literature about physical activity and mental health among refugees, (b) uses exercise and sport training as a first-line treatment (not as an add-on to usual care), (c) places a focus on (moderate-intensity) exercise activities, and (d) is implemented in a refugee camp before the refugees are relocated to a European host country.

## 2. Materials and Methods

### 2.1. Study Design

The present study is designed as a one-group, 8-week controlled intervention study including a pre-test/post-test design. The intervention took place between August and October 2017, in the Sinatex refugee camp, located in the north of Greece (Thessaloniki region), far away from neighbouring villages, in a former industrial area (for more details regarding the location of the refugee camp, see the footnote at the end of the manuscript). Due to the fact that the study was carried out without external funding, we were unable to intervene across a longer period of time. Moreover, our limited resources meant that it was impossible to include a waiting-list control group that would have received the intervention at a later stage. We therefore decided not to apply an randomized controlled trial (RCT) design, as we felt that it would be problematic, from an ethical point of view, to deny participants with severe mental health issues access to a potentially beneficial intervention. Instead, all interested participants had the opportunity to participate in the program. Participation was systematically assessed and considered as a potential predictor/moderator.

All procedures were carried out in line with the ethical standards laid out in the 1964 Declaration of Helsinki and its later versions. Moreover, all procedures were approved by the institutional review board of the medical association responsible for primary care at the camp. The researcher who carried out the intervention program (first author) signed the “Code of Conduct” of the Danish Refugee Council [28], including ethical principles such as respectful interaction with cultural particularities, fair and dignified dealings with all refugees, no preferential treatment of any individuals, maintenance of professional and emotional distance, not accepting any presents from the refugees in order to avoid conflict of interest, and keeping confidential information secret unless it is associated with a health risk for the person. As an incentive, participants received a bar of chocolate after each data assessment. This incentive was intended to attract less physically active and less motivated people, who might otherwise have avoided physical activity.

To determine the minimal sample size, we carried out a power analysis using G*Power software (version 3.1) (University of Düsseldorf, Düsseldorf, Germany). Rosenbaum et al. [9] found a moderate effect of exercise interventions on PTSD symptoms (Hedges *g* = 0.35). On the basis of Cohen’s [29] standards, our analysis showed that 43 participants were needed at the beginning of the study (linear multiple regression: fixed model; power = 0.80; alpha = 0.05; three predictors) to identify a moderate effect (*f*^2^ = 0.15). 

### 2.2. Participants and Procedures

All participants received detailed written and oral information about the study procedures and were asked to provide written informed consent before the start of the data assessment. We assured all participants that participation was voluntary, that they could withdraw at any time without having to justify their choice or fear that this would affect their status in the camp, and that nobody outside the research team would have access to their data. To ensure comprehension, some refugees with advanced English skills acted as interpreters during this process. 

Inclusion criteria were (a) informed written consent, (b) aged 16–49 years, (c) living in the Sinatex refugee camp (Greece), (d) being male, (e) not having any absolute contra-indication for moderate-intensity physical activity, and (f) being able to exercise three times per week. To assess contra-indications for moderate-intensity physical activity, participants were asked to complete the Physical Activity Readiness Questionnaire [30]. If contra-indications were present, a doctor was consulted before including a participant in the study. Exclusion criteria were (a) no written informed consent; (b) younger than 16 years, or older than 49 years; (c) not living in the Sinatex refugee camp; (d) not being male; (e) having an absolute contra-indication for moderate-intensity physical activity; and (f) having an injury that currently prevents the intended training load.

Taken together, the 45 participants provided written informed consent and took part in the baseline data assessments. However, 7 participants dropped out from the study, leaving a sample of 38 participants (dropout rate: 16%). Dropout was due to the fact that some individuals did not feel able to participate in the intervention, attempted to flee the camp, or were relocated by the government.

Among participants who completed both data assessments, 71% came from Syria, 16% from Iraq, 4% from Palestine, and 10% defined themselves as Kurds. In total, 74% of the participants were Muslims, 3% were atheists, and 23% did not provide information about their religious background. Most of the participants were between 16 and 30 years of age (*M* = 25.6, *SD* = 7.1).

All participants completed a questionnaire composed of several standardized and internationally validated instruments. The questionnaire was available in English and Arabic. 

Given that some participants had limited skills in these languages, we allocated sufficient time for the completion of the questionnaire, and carried out the data assessment in small groups (maximum of four participants) so that in case of unclarities the participants could discuss questions with the research officer and/or other participants with advanced language skills.

### 2.3. Exercise and Sport Program

Exercise and sport activities were chosen according to the preferences of the participants. The program was prepared in advance and presented to the participants to suggest adaptations. However, due to language barriers, the activities had to be simple and easy to understand. The program consisted primarily of the following activities: football, boxing, and a combination of weight and endurance training. Calisthenics, and weight training using old car/truck tires, sand-filled bottles, and big water bottles were core components of the program. However, other activities such as partner acrobatics, volleyball, and short hiking tours were included as well to make the program more varied. 

Activities were offered three (week 1–4; Monday, Wednesday, Friday) to five times per week (week 5–8; Monday to Friday). We chose to extend the number of training days in order to offer a broader range of activities. Whereas on Monday, Wednesday, and Friday, the focus was on weight and endurance training, on Tuesday and Thursday, we offered either football, volleyball, boxing, or partner acrobatics. This gave participants a certain choice to engage in activities they like. Moreover, there were no specifications regarding the intensity of the exercise and sport activities. Thus, participants were free to choose any intensity they liked. The duration of the exercise sessions was 60 minutes.

### 2.4. Measures

Symptoms of post-traumatic stress disorder. According to ICD-10 criteria, PTSD “arises as a delayed or protracted response to a stressful event or situation (of either brief or long duration) of an exceptionally threatening or catastrophic nature, which is likely to cause pervasive distress in almost anyone” (see https://icd.who.int). In the present study, we assessed PTSD symptoms with the 22-item Impact of Event Scale—Revised (IES-R) [31]. This instrument measures the severity of PTSD symptoms, with reference to the past 2 weeks. The instrument is internationally accepted, not culturally specific, and is available in several languages, including English and Arabic [32]. The IES-R items refer to DSM-V (Diagnostic and Statistical Manual of Mental Disorders, 5th edition) and ICD-10 criteria of PTSD. The IES-R has been used previously in refugee populations [33]. Items are answered on a five-point Likert scale from 0 (not at all) to 4 (extremely), resulting in an overall index between 0 and 88 points, with higher scores reflecting stronger PTSD symptom severity. The cut-off for a possible PTSD diagnosis is ≥33 [34]. The internal consistency of the IES-R overall index was good in the present study (Cronbach’s alpha = 0.92).

Depressive symptoms. We used the 9-item Patient Health Questionnaire (PHQ-9) to assess depressive symptoms [35]. Items of this instrument refer to DSM-V criteria for major depression. Answers are given on a four-point Likert scale ranging from 0 (not at all) to 3 (nearly every day), with reference to the past 2 weeks. The overall index varies between 0 and 27, with higher scores reflecting more depressive symptoms. Participants meet the criteria of major depression syndrome if (a) they report that they “have little interest or pleasure in doing things” or that they “feel down, depressed, or hopeless” more than half the days, and (b) indicate that they perceive at least five of the nine symptoms listed in the PHQ-9 more than half the days [35]. Given its brevity, the PHQ-9 is often used as a screening tool, and has been previously employed with refugee populations [36]. Evidence for the validity and reliability of the instrument with populations from the Middle East has been documented previously [37]. In our population, the internal consistency of the PHQ-9 was acceptable (Cronbach’s alpha = 0.77).

Anxiety symptoms. We used the 7-item anxiety subscale of the Hospital Anxiety and Depression Scale (HAD-A) to measure anxiety symptoms [38]. With reference to the last 7 days, items assess mood alterations as a consequence of anxiety states and are answered on a four-point Likert scale from 0 (not at all) to 3 (most of the time). Evidence for the validity and reliability of the HAD-A has been reported previously [39]. The cut-off for clinically relevant anxiety symptoms is ≥11 [40]. In the present study, the internal consistency was acceptable (Cronbach’s alpha = 0.73). 

Pain. We used a visual analogue scale (VAS) [41] to assess pain in several body regions (head, back, chest, stomach, upper and lower body extremities). Participants were asked to rate their current pain level. The VAS consists of a 10 cm horizontal line with two extremes (0 cm = no pain, 10 cm = pain as bad as it could be). The participants were asked to make a cross on this line, then the difference between point 0 and the cross was measured. A sum score was calculated across all body regions. Evidence of the validity and reliability of the VAS has been established previously [41]. In the present population, the internal consistency of the VAS pain scale was acceptable (Cronbach’s alphas = 0.71). 

Quality of life. Participants responded to the five-item WHO-5 (World Health Organization, 5-item) Index [42] to assess health-related quality of life. This instrument has been developed specifically for use in psychiatric populations [43]. With reference to the past 2 weeks, items are answered on a six-point Likert scale from 0 (at no time) to 5 (all the time). Items are summed up to generate an overall score between 0 and 25, with higher scores reflecting higher health-related quality of life. The instrument has been translated into more than 30 languages and used in numerous studies. The validity of the WHO-5 as a clinical screening tool has been demonstrated previously [44]. In the present sample, the internal consistency was acceptable (Cronbach’s alpha = 0.78). 

Self-perceived fitness. We used a 1-item measure to assess participants’ self-perceived fitness [45]. Participants are asked to rate their current fitness level on a Likert scale from 1 (poor) to 10 (excellent). Previous studies showed that self-perceived fitness is moderately associated with objective fitness measures [46]. Previous studies also showed that self-perceived fitness is more closely associated with subjective health perceptions than self-reported physical activity [47].

Cardiorespiratory fitness. We used the 20 m shuttle run test to measure participants’ cardiorespiratory fitness [48]. We explained the test procedures to the participants and demonstrated two trial runs. Once participants were familiar with the test procedures, they started with a running speed of 8.5 km/h on a premeasured running course on flat terrain, marked with colour-coded cones, following the primary investigator who set the pace according to an acoustic signal. We then gradually increased the frequency of the sound signal every min by 0.5 km/h. If participants did not reach the marked 2 m line at each end of the course for two successive intervals, they were asked to stop running and the fully completed laps were noted. Completed laps can be used to estimate maximal oxygen uptake (VO_2_max), and a recent meta-analysis shows that this estimate corresponds well with objectively assessed VO_2_max in adult populations [49]. 

Upper-body muscle strength. We used the grip strength test [50] as a measure for upper body strength. To do this, we used a hydraulic hand dynamometer (Saehan, MSD Europe BVBA, Tisselt, Belgium). The researcher gave a visual demonstration of how to hold the hand dynamometer. Participants were asked to sit down and assume a relaxed and upright position, with an arm position at a 90° angle. Every participant had four trials, alternating between the right and left hand with a 30 sec resting period between trials. All four trials were recorded to the nearest 1 kg and averaged. Fukumori et al. [51] showed that grip strength can be used as a predictor of future mental health.

Confounders. We assessed the following factors as potential confounders: age, body mass index (BMI; based on self-reported weight and height; kg/m^2^), nationality, religious background, educational background, time fleeing (in months), and time in camp (in weeks).

### 2.5. Statistical Analyses

We performed all statistical analyses with SPSS (Version 24, IBM, Armonk, USA). We used analyses of variance (ANOVAs) to examine whether dropouts and participants who completed the study differed with regard to social and demographic background and the primary and secondary outcome variables at baseline. Additionally, we used paired-samples *t*-tests to examine whether the mean scores of the primary and secondary outcomes changed across both measurement occasions. Finally, a series of hierarchical linear regression analyses were performed to discover whether baseline scores, participation rate, and the interaction between baseline scores and the participation rate predicted post-intervention scores in the outcomes. Variables were z-standardised before calculating the interaction term. Potential confounders were only considered if they were significantly associated with an outcome variable either at baseline or post-intervention. The level of significance was set at *p* < 0.05 across all analyses.

In total, very little missing data was identified (≤0.5%). Due to sickness or injury, five participants (13%) were not able to perform the handgrip test and seven participants were not able to perform the shuttle-run test (18%) either at baseline or follow-up. In the present study, all missing data were replaced via imputation, using an expectation maximization (EM) algorithm.

### 2.6. Data Availability

Data and intervention materials are available upon reasonable request to the corresponding author.

## 3. Results

### 3.1. Descriptive Statistics and Dropout Analyses

Baseline scores of participants who completed the study (*n* = 38) and dropouts (*n* = 7) are displayed in Table 1. Table 1 shows that two significant group differences existed at baseline, indicating that dropouts reported significantly more pain and had higher BMI scores than participants who remained in the study. Although no further significant group differences were found, the inspection of the effect sizes shows that the most vulnerable refugees were more likely to drop out. On a descriptive level, differences were found with regard to PTSD symptoms, depressive symptoms, anxiety symptoms, and sleep complaints, showing that dropouts had higher scores across all these variables; however, the differences were not statistically significant. Similarly, a small-to-moderate effect size was also found with regard to participants’ performance in the 20 m shuttle-run test, indicating that dropouts had considerably lower cardiorespiratory fitness levels than participants who completed the study.

Among participants who completed the study, 71% (*n* = 27) had a baseline score above the critical cut-off for PTSD (Creamer et al., 2002). At post-intervention, 55% (*n* = 21) of the participants exceeded this critical PTSD cut-off. At baseline, 26% (*n* = 10) of the participants met the criteria for major depression syndrome (Kroenke et al., 2002). This percentage slightly decreased at the post-assessment (18%, *n* = 7). Moreover, on the basis of the categorization of Zigmond and Snaith (1983), 42% (*n* = 16) of the participants exceeded the cut-off for clinically-relevant anxiety at baseline. At the post-assessment, this percentage dropped slightly to 34% (*n* = 13).

**Figure 1:**
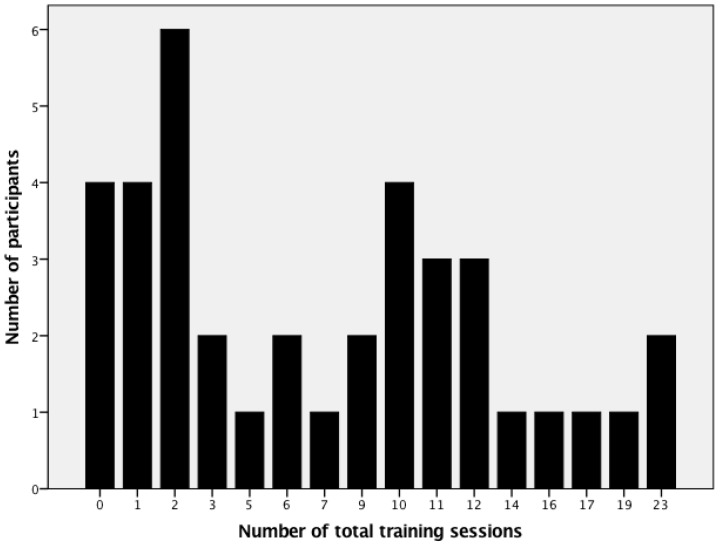
Distribution of total training sessions.

In total, 32 exercise sessions were offered across the intervention period. The mean participation rate in the exercise program was 7.5 exercise sessions (*SD* = 6.5). The distribution of training sessions across participants is displayed in Figure 1.

### 3.2. Associations between Confounders and Outcomes at Baseline and Follow-Up

Age was significantly related to PTSD symptoms at post-intervention (*r* = 0.39, *p* < 0.05). Age was also associated with performance in the shuttle run test, both at baseline (*r* = −0.34, *p* < 0.05) and post-intervention (*r* = −0.35, *p* < 0.05). Weeks of residence in the camp was correlated with depressive symptoms at baseline (*r* = 0.31, *p* < 0.05). No significant associations were found between the further confounders and outcome variables.

### 3.3. Changes from Baseline to Post-Intervention

Table 1 shows that the mean scores of the total sample (study completers) decreased significantly with regard to PTSD symptoms, depressive symptoms, and sleep complaints. By contrast, significant improvements were found for handgrip strength. A trend towards an improvement (*p* < 0.10) was observed for health-related quality of life and self-perceived fitness, whereas no changes were found for anxiety symptoms, pain, and performance in the 20 m shuttle run test.

### 3.4. Prediction of Post-Intervention Scores

Table 2 shows the inferential statistics of the outcomes. Across all hierarchical stepwise regression analyses, baseline scores significantly predicted post-intervention scores. More frequent participation in the exercise and sport program was associated with fewer anxiety symptoms at post-intervention. Moreover, more frequent participation predicted higher health-related quality of life, higher self-perceived fitness, better performance in the handgrip strength, and better performance in the 20 m shuttle run test. A non-statistically significant tendency also occurred for PTSD symptoms and depressive symptoms, indicating that more frequent participation in the exercise and sport program was associated with lower post-intervention scores in these two variables. Although not statistically significant, the regression coefficient pointed into the same direction for pain. No significant interactions were found between baseline scores and participation rate.

## 4. Discussion

The key findings of the present study are that refugees are a particularly vulnerable population, in which psychopathological symptoms are highly prevalent. This accords well with the findings of Madsen et al. [3], which suggested that two thirds of asylum seekers in Denmark met the ICD-10 criteria for PTSD. Our study also shows that among male refugees who live in precarious conditions in a Greek refugee camp, more frequent participation in an 8-week exercise and sport program has the potential to positively impact on their health. We are well aware that due to one group pre-test/post-test design and the lack of random assignment, the results of our study must be interpreted with caution. While randomized controlled trials are needed for an advanced evidence base, researchers should be aware that the implementation of such studies will be complicated by multiple knowledge barriers and environmental constraints [52].

Our study shows that more frequent participation was associated with better mental health across all variables. Significant main effects were found for anxiety and health related quality of life. However, due to the limited sample size, not all main effects for participation rate reached statistical significance. For PTSD symptoms and depressive symptoms, only statistically non-significant trends occurred. Taken together, our findings correspond well with previous reviews concluding that exercise interventions can contribute to the reduction of post-traumatic stress symptoms [8,9]. The uniqueness of our study results from the fact that our investigation is the first study with refugees living in a refugee camp, prior to relocation to other (European) countries. Our findings are also in line with investigations with refugees in host countries, showing that the promotion of physical activity has positive effects on refugees’ health [2,3,15,25]. Meanwhile, several organizations highlight that there is a great need to improve the mental health of refugees [21]. Early interventions are needed to avoid the entrenchment of mental disorders, which may hinder the integration process once refugees are officially accepted as refugees in the host countries [22].

The fact that frequency of participation in an exercise program tends to have a positive impact on a variety of health outcomes is also in line with previous research. Meanwhile, a multitude of studies exist showing that exercise and sport interventions can contribute to the prevention and therapy of mental health issues such as major depression [26,53], anxiety disorders [54], and pain [55]. Furthermore, it is well established in non-refugee populations that exercise and sport have a beneficial impact on participants’ health-related quality of life [27]. Although not the focus of the present study, it has been suggested in previous research that exercise-related improvements in mental health can be attributed to a number of different factors such as exercise-related secretion of noradrenalin, serotonin or brain-derived neurotrophic factor [56], repeated occurrence of positive mood states [57], improved coping with stress [58], or psychological factors such as satisfaction of basic psychological needs [59], and improved self-perceptions and perceived competence regarding one’s body and its functioning [60].

It is also noteworthy that in our study regular participation in the exercise and sport program was associated with better cardiovascular fitness at post-intervention. This finding is important because low fitness levels are associated with increased risk for cardiovascular and other chronic diseases [61], particularly among people who feel exposed to high chronic stress levels [62]. The findings of our study are also in line with previous research showing that exercise training effectively contributes to the improvement of physical fitness in patients with psychiatric disorders [63]. On the other hand, previous research has shown that regular fitness training has the potential to reduce many cardiometabolic risk factors such as overweight/obesity [64], blood pressure [65], diabetes [66], unfavourable blood profile [62], and metabolic syndrome [67]. Regular exercise training also has the potential to prevent osteoporosis [68]. Accordingly, as refugees have higher odds of developing such diseases [69,70], exercise and sport programs are likely to mitigate some of these cardiometabolic risks. Finally, regular participation in the exercise and sport program was associated with higher post-intervention scores with regard to upper-body strength, as assessed via the handgrip strength test. This finding deserves emphasis as evidence is growing that higher handgrip strength is associated with better mental wellbeing [71].

Another important outcome of our study is the fact that more frequent participation in the exercise and sport program positively predicted self-perceived fitness. Previous research has shown that perceived fitness is closely associated with mental wellbeing [47]. Furthermore, it is likely that increased self-perceived fitness goes along with increased self-efficacy to engage in exercise and sport activities [72]. As highlighted in the introduction, mental disorders are often paralleled by a mind-set that may interfere with the adoption and maintenance of a physically active lifestyle (e.g., feelings of exhaustion, hopelessness, resignation, social withdrawal) [73]. Therefore, improving self-perceived fitness might be an important starting point for refugees to initiate the adoption of a more physically active lifestyle. 

The strengths of the present study were that we were able, for the first time, to evaluate the impact of an exercise and sport program with refugees living under difficult life circumstances in a refugee camp. Another strength was that the exercise and sport program was designed together with the participants, in order to take into account the preferences of the target group. Moreover, the participants were allowed to freely chose the days on which they exercised and the number of sessions they participated in. Despite a limited participation rate, on the basis of a single-item question (“How satisfied were you with the contents of the exercise program?”), 50% of the participants reported that they were satisfied, 45% very satisfied, whereas only 5% were not satisfied. 

Considering the socio-political situation in Europe following the recent influx of refugees and the importance of exercise as a health and socio-cultural agent, the topic of the present study is timely and innovative. Although we believe that our study can help opening the field, some aspects need to be considered that may limit the generalizability and impact of our findings. First, only male participants took part in our study (mainly younger adults). Thus, it remains unknown if similar effects would have occurred among women and younger/older participants. One study has looked at some similar issues among women immigrants and refugees in the United States of America [74]. Second, although the chosen activities were in the moderate-to-vigorous range [75], intensity levels were not systematically assessed during the training sessions. Accordingly, it was not possible to consider energy expenditure as a possible moderating or confounding factor. Another potential confounder is that we did not systematically assess independent-of-the-programme physical activity. Moreover, the post-test took part under different climatic conditions than the pre-test. That is, the outside temperatures were considerably cooler. Accordingly, the participants were likely to have sweated less during the fitness test, and may consequently have had a better grip when using the hand dynamometer. This may have had an influence on participants’ performances in the shuttle run test. We also acknowledge that the participation rate in the program was relatively low, which might have negatively affected the impact of the program. Thus, researchers should examine in future research if participants benefit more if they accomplish “public health doses” [76]. Another critical point is that we did not systematically assess substance consumption and/or consumption of psychopharmacological drugs. Accordingly, we cannot exclude that antidepressants (or other drugs) were given to the participants during the course of the intervention and that this contributed to their improvement.

Furthermore, the fact that a pre-experimental design (one group pre-test/post-test study) was used needs to be discussed critically. Our decision was based on the fact that we did not have the resources to include a waiting-list control group. Therefore, we found it ethically problematic to purposely withhold a potentially beneficial intervention to refugees willing to participate in the program. Using a pre-experimental design complicates causal conclusions for several reasons. For instance, one might argue that participants with lower participation rates generally perceived more psychological strain. However, this issue did not seem to apply to the present sample, as participation rate was not significantly correlated with any of the mental health outcomes at baseline. Nevertheless, we cannot exclude the possibility that critical life events occurred during the intervention period, which may have contributed to the fact that some participants felt less able to continue their engagement in the exercise training program. One could therefore argue that the lower mental health scores of participants with lower participation rates may be attributable to higher exposure to critical life events. However, this assumption remains speculative, as we did not collect information about life events during the intervention period.

Our study also shows that it is difficult to motivate the most vulnerable refugees for an exercise program. As shown in Table 1, dropouts reported considerably higher symptoms of mental ill-health across all indicators. Most importantly, dropouts perceived substantially higher pain levels than the rest of the study participants. For some refugees, somatic health problems might have been so strong that participation in an exercise programs seemed not possible. Therefore, further investigation is required to determine what forms of physical activity these individuals would feel able to engage in. Alternatively, it can also be argued that the shuttle run test is a strenuous test, which might have discouraged the most vulnerable individuals from participation. Thus, for future research it might be recommendable to use submaximal fitness tests in order to reduce the number of dropouts. 

As mentioned previously, more randomized controlled trials are needed to establish relationships between cause and effect. However, carrying out such studies in a refugee camp is challenging for several reasons. First, it is difficult to find sufficient participants willing to take part in such a study, and to be randomly assigned to a control group. Second, to keep participants motivated, it is important for the exercise coach to build up a relationship of mutual trust and confidence. Accordingly, the influence of experimenter effects needs to be considered carefully. Third, it is difficult to create controlled experimental conditions. Daily-life influences (e.g., conflicts between different ethnic groups in the camp) or major life events (e.g., negative asylum decision) can override or offset the positive influences of an exercise trial. Fourth, few refugees are used to participating in scientific studies, and limited language skills may complicate the completion of self-report questionnaires. As described in the method section, we have addressed this issue by providing two language versions of our questionnaire, organizing the data assessment in small groups, allocating sufficient time for answering the questions, and providing support to those participants with limited language skills either through the research officer or other participants with more advance language skills. Fifth, a substantial dropout rate needs to be expected because a considerable number of participants may withdraw their informed consent or disappear before the post-assessment has taken place (e.g., due to escape from refugee camp, relocation to host-country). In our sample, the dropout rate was 16%. As dropouts had lower scores in the 20 m shuttle run test, one could speculate that this (maximal) fitness test might have been a negative experience for them. However, cross-sectional correlations between fitness parameters and the other study variables showed that with the exception of age, performance in the 20 m shuttle run test was not statistically significantly associated with any of the other study variables. It should also be noted, however, that psychological variables (PTSD symptoms, depressive symptoms, etc.) showed a trend-level inverse association with performance in the 20 m shuttle run test. Taken together, much more could be written about the challenges that we faced when carrying out our study. However, this would go beyond the discussion of our (quantitative) findings. Nevertheless, as many lessons can be learned from our experiences, we are currently preparing a (qualitative) report to facilitate the planning and implementation of future empirical trials in a refugee camp context.

## 5. Conclusions

This study is the first to implement an exercise and sport program in a refugee camp, and therefore represents a significant step forward for the field. More frequent participation in an 8-week exercise and sport intervention in male camp residents was associated with better mental health and higher self-reported and objectively assessed fitness at post-intervention. This is in line with the large evidence base suggesting the physical activity can impact positively on psychiatric health. In view of the numerous calls in the literature for rapid interventions to improve the mental wellbeing of refugees, this study is a first indicator that exercise and sport are both feasible and effective. Subsequent programs must attempt to perform an ongoing assessment of significant life events and stressors, in order to determine whether these have an impact on compliance; adapted physical activity for refugees with physical limitations must also be thoroughly explored.

## Figures and Tables

**Table 1:** Group differences in socio-demographic background between dropouts and refugees who participated in both data assessments at baseline, and post-test scores of participants.

	Baseline: Total Sample (*n* = 45)	Baseline: Dropouts (*n* = 7)	Baseline: Participants (*n* = 38)	Test of Group Differences at Baseline		Post: Participants (*n* = 38)	Test of Changes from Baseline to Post-Test		
	*M (SD)*	*M (SD)*	*M (SD)*	*F*	η^2^	*M (SD)*	*t*	*d*	*95% Confidence Intervals*
Social and demographic background									
Age	25.3 (6.8)	23.5 (4.0)	25.5 (7.1)	0.47	0.011	---	---	---	
BMI	22.2 (3.5)	24.9 (4.6)	21.8 (3.2)	4.12 *	0.089	---	---	---	
Years of education	9.5 (3.2)	10.0 (1.8)	9.6 (3.3)	0.10	0.002	---	---	---	
Months since fleeing home country	34.0 (40.9)	22.5 (25.0)	32.9 (39.4)	0.40	0.009	---	---	---	
Weeks in camp	20.8 (18.1)	26.4 (20.3)	20.0 (18.2)	0.47	0.013	---	---	---	
Primary and secondary outcomes									
PTSD symptoms	48.1 (21.2)	60.7 (16.2)	45.7 (21.5)	2.65	0.059	35.4 (25.5)	3.53 **	0.57	(0.23 to 0.91)
Depressive symptoms	11.9 (6.3)	16.3 (7.3)	11.1 (6.0)	3.80 ^+^	0.083	9.2 (6.2)	2.48 *	0.40	(0.07 to 0.73)
Anxiety symptoms	9.6 (4.2)	12.2 (4.0)	9.1 (4.2)	2.76	0.062	8.2 (5.0)	1.53	0.25	(−0.08 to 0.57)
Sleep complaints	14.1 (6.0)	17.8 (5.9)	13.4 (6.0)	2.88 ^+^	0.064	11.1 (7.6)	2.11 *	0.34	(0.01 to 0.67)
Pain	128.3 (94.6)	216.8 (130.4)	109.0 (74.9)	8.64 **	0.171	91.9 (72.6)	1.55	0.25	(−0.07 to 0.57)
Health-related quality of life	8.1 (5.9)	5.3 (4.7)	8.6 (6.0)	1.61	0.037	10.6 (6.8)	−1.72 ^+^	−0.28	(−0.60 to 0.15)
Self-perceived fitness	5.0 (2.2)	4.8 (3.3)	5.1 (2.1)	0.05	0.001	5.3 (2.0)	−1.07 ^+^	−0.17	(−0.49 to 0.15)
Handgrip strength test	41.0 (6.7)	42.4 (9.2)	41.0 (6.2)	0.24	0.006	44.9 (6.7)	−5.61 ***	−0.91	(−0.53 to −1.28)
20 m shuttle run test	46.6 (16.1)	37.8 (4.4)	48.3 (16.9)	2.24	0.051	50.7 (19.0)	−1.34	−0.21	(−0.54 to 0.11)

*Notes.* PTSD = Post-traumatic stress disorder; ^+^ *p* < 0.10, * *p* < 0.05, ** *p* < 0.01, *** *p* < 0.001.

**Table 2:** Hierarchical stepwise regression analyses to predict post-intervention scores.

	**PTSD Symptoms**		**Depressive Symptoms**		**Anxiety Symptoms**		**Sleep Complaints**		**Pain**	
	**β**	**Δ*R*^2^**	**β**	**Δ*R*^2^**	**β**	**Δ*R*^2^**	**β**	**Δ*R*^2^**	**β**	**Δ*R*^2^**
Step 1		0.533 ***		0.536 ***		0.415 ***		0.298 ***		0.334 ***
Age	0.14		---		---		---		---	
Weeks in camp	---		0.07		---		---		---	
Baseline score	0.67 ***		0.73 ***		0.65		0.51 ***		0.56 ***	
Step 2		0.036 ^+^		0.042 ^+^		0.036 ^+^		0.040		0.041
Participation	−0.19 ^+^		−0.29 ^+^		−0.27 *		−0.21		−0.20	
Step 3		0.001		0.002		0.002		0.022		0.022
Baseline score × Participation	0.03		0.04		−0.05		−0.15		−0.18	
	*R*^2^ = 0.570 ***		*R*^2^ = 0.580 ***		*R*^2^ = 0.487 ***		*R*^2^ = 0.360 ***		*R*^2^ = 0.397 **	
	**Health-Related Quality of Life**		**Self-Perceived Fitness**		**Handgrip Strength Test**		**20 m Shuttle Run Test**			
	**β**	**Δ*R*^2^**	**β**	**Δ*R*^2^**	**β**	**Δ*R*^2^**	**β**	**Δ*R*^2^**		
Step 1		0.155 *		0.444 ***		0.612 ***		0.676 ***		
Age	---		---		---		−0.05			
Baseline score	0.40 **		0.62 ***		0.76 ***		0.75 ***			
Step 2		0.083 ^+^		0.098 **		0.048 *		0.043 *		
Participation	0.32 *		0.32 **		0.23*		0.22 *			
Step 3		0.034		0.001		0.002		0.002		
Baseline score × Participation	−0.19		−0.04		−0.04		−0.04			
*R* ^2^	*R*^2^ = 0.272 *		*R*^2^ = 0.543 ***		*R*^2^ = 0.662 ***		*R*^2^ = 0.721 ***			

*Notes.* PTSD = Post-traumatic stress disorder; ^+^ *p* < 0.10, * *p* < 0.05, ** *p* < 0.01, *** *p* < 0.001.
